# Refugee women’s experience of the resettlement process: a qualitative study

**DOI:** 10.1186/s12905-019-0843-x

**Published:** 2019-11-27

**Authors:** Elisabeth Mangrio, Slobodan Zdravkovic, Elisabeth Carlson

**Affiliations:** 1 Department of Care Science, Faculty of Health and Society, Malmö University, Malmö, Sweden; 2 Malmö Institute for Studies of Migration, Diversity and Welfare (MIM), Malmö University, Malmö, Sweden

**Keywords:** Migration, Resettlement, Qualitative research, Women

## Abstract

**Background:**

Resettlement can be particularly challenging for women as having a lower socioeconomic status and language barriers, may impede women’s access to education, employment opportunities, health-care services, as well as the cultural, social, material and resilience factors that facilitate adjustment and adaption. Thus, the aim of this study is to further explore the perception of refugee women in Sweden concerning their situation during active participation in the resettlement process in the country.

**Methods:**

Qualitative interview study with 11 recently arrived refugee women who had received their residence permits and were enrolled in the resettlement process. The interviews were conducted in Swedish with the support of an authorized Arabic translator present by telephone.

**Results:**

Refugee women suffered from being separated from their loved ones and felt compelled to achieve something of value in the host country. All experienced both physical and mental anguish.

**Conclusions:**

Stakeholders in societies that receive refugee women should stress the importance of finding opportunities for and fast entrance into employment in the host countries. This would be beneficial for the integration and well-being of refugee women after migration.

## Background

All refugees, independent of sex, have significant needs during their resettlement in recipient countries. However, women’s gendered experiences both pre- and during flight from areas of global conflicts, combined with encountered stressors in exile, result in their needs being different from those of refugee men [1, 2]. Refugee women are to a higher extent vulnerable to physical and mental health difficulties [3] and flight is often more difficult for women than for men as they are more vulnerable to sexual assaults and exploitation by armed forces [1]. Refugee women’s experiences include exposure to violence, and unique physical, mental and social needs, as well as various health related problems. Further, the wellbeing of women after flight is closely connected to how well their children are feeling, adapting and performing [4, 5]. In addition, lower socioeconomic status and language barriers contribute to the resettlement process being particularly challenging for women. This could impede their access to education, employment opportunities, health-care services, as well as access to cultural, social, material and resilience factors that facilitate adjustment and adaption [6].

Over the last years Sweden has received a large influx of refugees. In 2015, 160,000 refugees, mainly from Syria, Iraq and Afghanistan, came to Sweden [7]. Since 2015, the number of refugees has declined due to a restrictive immigration policy. By 2017 approximately 25,000 refugees were received by Sweden, decreasing to 21,000 in 2018 [8].

All refugees that have received asylum status are enrolled into the establishment process based on an individually developed introduction plan. The employment service in Sweden is in charge of this program, which provides the refugees with approximately 2 years of support including access to Swedish language programmes, courses on preparation for employment as well as civic and societal information. The aim of this plan is to offer support to facilitate integration into the labour market as soon as possible. This is achieved by tailoring the introduction program to capitalize on the individual refugee’s skills, such as education and work experience [9].

Regarding their situation in the resettlement process in Sweden and their perception of health, social relations, employment and housing [10], refugees encounter issues of crowded living environments, low confidence in health care services, and mental illness. Self-reported health among the refugees in Sweden is considered to be in line with the rest of the Swedish population, but higher levels of obesity/being overweight, smoking and physical inactivity are reported compared to the rest of the population in Sweden [10]. In a previous study [10] refugee women responded to a health survey to a lesser degree than men. Considering the gender specific challenges during the flight, the current study aims to explore refugee women’s perception of their situation and active participation during the resettlement process in Sweden.

## Methods

A qualitative study was conducted through interviews with recently arrived refugee women who had received their residence permit, with an introduction plan [11] and enrolled in the resettlement process. Throughout the research process we adhered to the COREQ guidelines in order to promote complete and transparent reporting and improve the rigor, comprehensiveness and credibility of the interview study [12].

### Selection process

The first author worked together with the civic and health communicators from the provincial government [13] who assisted in recruiting informants for the study. The invitation to participate in an interview was done in conjunction with the regular meetings that civic and health communicators had with newly arrived refugee women. In total, approximately 100 women were approached through convenience sampling. They received information about the study and were asked to participate during the civic and health communication. Of the 100 women approached, 11 volunteered to participate. The process that followed included a formal invitation by telephone for study participation. None of the authors had any relationship with or knowledge about the interviewed women prior to the interviews. Before the interviews took place, the researcher gave both oral and written information about the study as well as how all the data collected during the interviews, was to be anonymized and stored securely. Before the interviews, all interviewees had to sign an informed consent form.

### Data collection

All written information circulated to the tentative informants was translated by authorized translators. The location for interviews was either at the participant’s home or at the school for the Swedish studies. An interview guide that covered different topics regarding the family’s situation and being in the resettlement process was used during the interviews.

(Additional file 1). The first author (EM) conducted all interviews and is an experienced qualitative researcher with prior experience of interviewing persons with different ethnic backgrounds. The interviews started with a broad question inviting the women to talk about the experience of arriving to and living in Sweden. Thereafter, the interview covered topics such as how the women perceived their situation, their health, the struggles they face and their hopes for the future. The interviews were conducted in Swedish and with Arabic translation. The Arabic translation was conducted over the phone. The translators were of mixed gender and were authorized. All interviews were recorded and transcribed verbatim. A total of 11 recently arrived refugee women participated in the interviews. After these interviews, no new information emerged; thus, the data collection was terminated. The interviews averaged 30 min (17–48) including interpretation. The age of the interviewees averaged 34 years (25–50) and nine out of eleven had an educational level above 12 years. The interviewees had three children on average (one -seven) and all were married.

### Data analysis

First, the transcripts were read, reread and coded by the first author. Second, the coded material was read by the last author and feedback about the coding ensured credibility. The material was analysed with content analysis, following the method by Burnard [14]. Table 1 provides excerpts from meaning unit, subcategory to category.

1. Interviews were read and transcribed verbatim in either Swedish or in English.
2. Open coding was performed and used to create sub-categories.
3. After clarifying the meaning of each subcategory, they were grouped into categories.
4. The results were discussed by all three authors and adjustments were made continuously until all data were used.

**Table 1:** Excerpts from meaning unit, subcategory to category

Meaning unit (extracts from the interviews	Sub category	Category
“I miss my parents that are left in Iraq” (9)	A sense of loneliness	Suffering from being separated from loved ones
“try to know what kind of people are here and in what way can I communicate more with them so that I learn Swedish” (1)	The importance of learning the language	To live with pressure to achieve the best possible life
“I have suffered from a terrible headache; and during 1 month, I was bedridden because of pain and was not able to do anything”	To regain physical health	Balancing health and illness

## Results

The results were divided into three categories and associated sub-categories: Suffering from being separated from loved ones, to live with pressure to achieve the best possible life and balancing health and illness.

### Suffering from being separated from loved ones

During the interviews, many of the women commented on the struggle they had faced concerning being separated from other family members. In addition, they reported their emotional reactions to these separations and their subsequent feelings of loneliness. The following sub-categories emerged from this category: *Reactions to being split as a family* and *a sense of loneliness.*

### Reactions to being split as a family

One woman spoke of her father’s passing just before she left for Sweden and conveyed that she was worried about the rest of the family left suffering in Syria. Similarly, another related her concerns about her being separated from her family, as illustrated below:

*“I am not feeling well since my parents are still in Syria suffering from physical ailments, and I am worried as the situation in the country is not secure”* Informant 10.

A different respondent revealed that she had not seen her brother and sister for 8 years; and because they were in Germany and Norway, they were unable to meet. Another with parents and siblings still in Syria wished they were able to flee to Sweden; however, she did not consider this a possibility.

### A sense of loneliness

Several women expressed feelings of loneliness due to being separated from parents or extended family members. They explained that the loneliness caused them to have heart ache that was difficult to bear. Those women, who were without any relatives in Sweden, not only felt lonely but also wondered how they would be able to start a new life here without them. One woman spoke about the emotions this evoked, wondering how she would be able to manage alone. A different woman captured the situation when she stated:

*“Since I missed my parents and my sisters and brothers when I came to Sweden, you feel like there is something in your life lacking”* Informant 9.

### To live with pressure to achieve the best possible life

This category emerged from almost all interviewed women, many of whom related their desire and willingness to achieve something through both studies and employment after their arrival in Sweden. They also raised the importance of learning the Swedish language well. Additionally, the women reported setting new goals for their lives here, while trying to balance and combine different aspects of their life. The following sub-categories emerged after the analysis: *Wanting to achieve something, setting new goals, the importance of learning the language* and *to be able to combine different aspects of life.*

### Wanting to achieve something

Several women communicated that they wanted to do their best and really achieve something of value in Sweden. As one woman noted:

*“We will study, and we will work hard to serve this community and society”* Informant 1.

Another related that she had registered for a course and was simultaneously applying for employment. A well-educated interviewee had high hopes of finding a job matching her educational level. However, she was offered only a position as a nanny, which she found disappointing. Other women shared their intention to complete the studies they had started in their home country. One woman articulated this desire, saying:

*“When I am done with my Swedish studies, I will continue to university studies; and I want to become a teacher of geography”* Informant 10.

### Setting new goals

The women spoke not only of setting new goals after arriving in Sweden but also of how the planned to reach them. However, other women were disappointed with themselves for not achieving their goals to secure employment. One woman reflected on the goals that she had set herself:

*“Even if you know other people here, everyone is fighting for themselves and towards reaching their individual goals. The future will be easier if the goals could be reached”* Informant 8.

Though one interviewee had planned to attend university, this materialized as something which would take many years, as she first had to undergo pre-university studies. Another had prioritised the continuation of her studies in Sweden.

### The importance of learning the language

During the interviews, the women spoke of their intention to learn the Swedish language well enough to be able to continue studying in the country. To learn Swedish quickly, one respondent practiced newly acquired words each evening and read books in Swedish regularly. Moreover, the women stressed the importance of learning the language so as to be active in society. As revealed by one respondent:

*“If you don’t learn the language, it won’t work. So, the responsibility is on us”* Informant 6.

For other women, learning Swedish was a means to be more independent and to open doors for new possibilities. Learning the language fluently was also highlighted.

### To be able to combine different aspects of life

Another important aspect that came to the fore was how the women strived to balance the workload of different activities both at school and at home with regard to taking care of their children. Though this sometimes was stressful, the women communicated they could manage on the whole. A woman who also had elderly parents to take care of explained:

*“It has become less frequent no; but in the beginning in Sweden, I had to help my parents a lot and at the same time manage my schedule in school. But it all worked out well”* Informant 4.

Some women spoke of prioritizing their small children and giving them a lot of attention, reasoning that children needed their mothers more than the women needed to study, with the latter being something they could focus on when the children were a little bit older. Nonetheless, others maintained that one has to combine home and studies to achieve something in Sweden.

### Balancing health and illness

This category emerged from almost all the interviewed women, through their accounts of struggling with various physical ailments and mental suffering. Keeping the balance between health and illness was perceived as a challenge. The following subcategories emerged after the analysis: *To regain physical health* and *to stay mentally fit.*

### To regain physical health

The interviewed women talked about the different kinds of health issues they faced, such as high blood pressure, headaches, bodily pain and gynaecological issues, to mention a few. Some revealed how the health issues they had before their flight to Sweden had gotten worse after arrival. One woman explained her struggle:

*“I have suffered from a terrible headache; and during one month, I was bedridden because of pain and was not able to do anything”* Informant 7.

For this woman, headaches affected her ability to think clearly during her studies and work. Another woman recounted her daily struggle with leg-pain. Though doctors had informed her this would soon disappear, this was not the case. Despite the pain, she had to walk to collect her children from school every day.

### To stay mentally fit

Regarding the mental struggles the women encountered upon arriving in Sweden, many instances of this were related to the interviewer. For one respondent, not being able to walk by herself due to not knowing the language or the country was particularly stressful:

*“In the beginning, I needed someone that could help me communicate and show me where to go and that stressed me and made me suffer mentally; and therefore, I isolated myself even more”* Informant 2.

The same woman reflected on the mental anguish she suffered when her application to stay in Sweden was denied. Another cause of stress for the women was the realization that family income would be reduced, thereby adding the necessity of finding employment. Others felt anxious when faced with the difficulty of finding suitable employment.

## Discussion

The results revealed that the recently arrived refugee women in Sweden suffered from being separated from their loved ones and from the pressures of wanting to achieve something of value in the host country. Being separated from family members after arrival caused different kinds of emotional reactions, such as loneliness. This is in line with earlier research, which reveals that there are beneficial effects of the presence of family after a person or family has fled as refugees [15–17]. It is also known that mental well-being is essential for an effective integration [11]. Therefore, it could be of importance for stakeholders in receiving countries to consider the impact of family reunification for newly arrived refugees.

Many women participating in the present study expressed a willingness to contribute to society and emphasised the importance of studying the language in order to find employment or to pursue university studies. It is already known that learning the language of the host country is a key factor for successful integration [18]. Organizations such as churches, NGOs, multicultural support groups and literacy centres have been shown to be good facilitators for improving language skills of refugees during resettlement [19]. Consequently, stakeholders working with resettlement in different societies should encourage refugees to seek out such meeting points. Based on this study, as well as earlier research [18], we stress the importance of learning the language to more quickly enter into employment. The women also showed a willingness to engage in preparation for employment. It is vital to involve recently arrived refugees in the labour market, as unemployed women have poor self-rated health and higher rates of psychological distress [20].

The women included in this study also raised physical and mental complaints. Feeling well both physically and mentally is crucial if migrants are to actively participate in the establishment process [11]. Health is not just a human right but also a presumption for being fully active in society. Because good health is a prerequisite for employment and integration into society, it is thus a foundational factor for establishing social relations in a new society [11]. However, a recently published Swedish study stated that health literacy, which means an ability and motivation to gain access to, understand and use health information, is lower among refugees compared to the general population [21]. They also saw that migration into a new country entailed obstacles including communication as well as learning to navigate a new health system. The civic and health communicators were perceived as a link to the new country and a source of information about the health care system [21]. Another recently published study in Sweden focusing on health care experiences among recently arrived refugees revealed that over 70% of participants had been in need of health care during the last 3 months but not sought care [22]. The reasons for not seeking care were high costs, long waiting times and language difficulties [22]. Both lower health literacy as well as difficulties to obtain health care for newly arrived refugees are important factors to consider; since good health is important for the integration of refugees in Sweden [11]. It is also important to continue the civic and health information given to all newly arrived refugees, in order to build links between the refugees and the new society in Sweden [21].

Almost all interviews were conducted in Swedish with Arabic translation simultaneously by telephone. We are well aware that the use of translators could lead to power imbalances [23]. However, we argue that this was not a problem in the present study since translation was done by phone. Therefore, we consider that the risk of such an error is minimal, since the translators were not physically present and thus less prone to interfering during the conversation between the researcher and the interviewees. However, we could not totally eliminate that risk.

With the interviews being conducted in Swedish, with Arabic translation, and then the findings being presented in English, we are aware of the risk of some information being lost in translation [23]. In addition, it might be considered as a limitation of the study that the informants were approached through a convenience sampling technique. While the research does not claim to draw on a representative sample of the population, it is argued that this approach does provide a useful empirical lens through which to understand refugee women’s experiences of resettlement.

Among these 11 interviewees, nine had a higher educational level (above 12 years) which is higher than the average among refugees in Sweden [24]. This fact needs to be considered regarding the transferability of the study [25].

## Conclusions

It is important to reunite refugee families because separation could affect mental well-being after arrival. Recently arrived refugee women seemed to be eager to enrol themselves in Swedish studies and to find employment. These are beneficial qualities which are highly relevant when considering in the resettlement process. Consequently, stakeholders in societies that receive migrants should stress the importance of finding opportunities and fast entry into employment in the host country. This would be beneficial for both integration as well as the well-being of refugee women after migration.

## Supplementary information

**Additional file 1.** Interview guide.

## Data Availability

Pursuant to national legislation, ethical review boards in Sweden do not allow release of sensitive raw data to the general public. Excerpts from the interviews have been translated and are a part of the results.

## Abbreviations

EC: Elisabeth Carlson
EM: Elisabeth Mangrio
SZ: Slobodan Zdravkovic
