# Supplementary material for: Refugee women’s experience of the resettlement process: a qualitative study
Source: BMC Womens Health. 2019 Nov 27;19:147. doi: 10.1186/s12905-019-0843-x (PMC6882316; doi:10.1186/s12905-019-0843-x)
Supplement: Supplementary file 1 — Additional file 1. Interview guide. [file 12905_2019_843_MOESM1_ESM.docx]

| **Interview guide** |
| --- |
| Can you please tell me about your escape to Sweden?  How do you currently experience your situation?  How is your physical, mental and social health?  How are your children doing?  Can you tell me what you think of your experience of the escape?  What challenges do you face as a family?  What possibilities for the future do you perceive for your family? |
